# A System of Dental Surgery

**Published:** 1887-05

**Authors:** 


### Bibliographical.

---

A SYSTEM OF DENTAL SURGERY.—By Sir John Tomes, F. R. S. Third Edition. Revised and enlarged by Charles S. Tomes, M. A., F. R. S., with 292 illustrations. Publishers: P. Blakiston, Son & Co., Philadelphia, 1887.

This old and well-known work in the form of a new edition, has been recently published, in which some of the sections of the former edition have been omitted and other new ones added, comprising thirty pages and twenty-nine illustrations. While this work might not meet the requirements of the dental student as a text book, on account of its not being comprehensive enough for such a purpose, yet there is much useful imformation to be found on its pages, which will prove valuable to every member of our profession. Some fourteen years have elapsed since the appearance of the second edition which, on account of the rapid and material development of the science of dentistry, has rendered necessary considerable revision and change. We still notice the retention of old and obsolete words, which are at variance with the improved nomenclature of our literature.

The portion of the work relating to dental histology has been somewhat changed to conform to the latest investigations, but not to the extent required by recent discoveries. The inflammatory or vital theory of the causes of dental caries is yet adhered to, although the solvent action of acids is admitted to a certain degree. The portion of the work treating of irregularity of the teeth is perhaps more advanced than any other, although the unsafe practice of torsion is yet advocated. The various forms of irregularity, however, with their causes and phenomena are ably treated. A very considerable portion of this work is devoted to oral surgery, and the treatment recommended for devitalized teeth is by no means encouraging. On the other hand the treatment of exposed pulps and the use of plastic materials for filling teeth present some valuable suggestions, and care is observed to avoid the extremes of conservative treatment. The concluding portion of the work is devoted to some methods of pivoting illustrated by cuts, and constitutes a valuable, though brief chapter. American methods are explained and generally recommended throughout the entire portion of the work relating to practical matter.

